# Exercise-Induced Functional Changes in People with Parkinson's Disease following External Cueing and Task-Based Intervention

**DOI:** 10.1155/2024/6188546

**Published:** 2024-01-19

**Authors:** Christine M. Clarkin, Christie L. Ward-Ritacco, Leslie Mahler

**Affiliations:** ^1^Physical Therapy Department, University of Rhode Island, Kingston, Rhode Island, USA; ^2^Interdisciplinary Neuroscience Program, University of Rhode Island, Kingston, Rhode Island, USA; ^3^Department of Kinesiology, University of Rhode Island, Kingston, Rhode Island, USA; ^4^Department of Communicative Disorders, University of Rhode Island, Kingston, Rhode Island, USA

## Abstract

**Introduction:**

The purpose of this study was to evaluate change in motor function, gait speed, dynamic balance, balance confidence, and quality of life (QoL) in nine participants with Parkinson's disease (PwPD) completing Lee Silverman Voice Treatment BIG (LSVT-BIG), an external cueing and task-based intervention. Although supported as an efficacious treatment in PwPD, there is limited research examining clinically meaningful change in outcome measures related to external cueing and task-based interventions.

**Materials and Methods:**

This was a case series of nine PwPD (age range 64-76 years, 55% male) who completed the LSVT-BIG protocol. Disease duration ranged from 1 to 17 years and was classified as moderate in all participants (Hoehn and Yahr = 2 or 3). Outcome measures included motor function (MDS-UPDRS Part III Motor), gait speed, dynamic balance (MiniBEST), Activities-specific Balance Confidence (ABC), and Summary Index for PD Quality of Life 39 (PDQ-SI). Assessments were completed at baseline (BASE), end of treatment (EOT), and 4 weeks after EOT (EOT+4).

**Results:**

Minimal detectable change (MDC) or minimal clinical important difference (MCID) was observed in one or more outcome measures in 8 of 9 participants at EOT and EOT+4 across domains of motor function (67%, 78%), gait speed (78%, 67%), balance confidence (44%, 33%), quality of life (44%, 78%), and dynamic balance (22%, 22%). *Discussion*. In this case series, 8 of 9 participants showed MDC or MCID changes across multiple functional domains. Improvements were observed immediately post (EOT) and 4-week post-treatment (EOT+4) suggesting a temporal component of the LSVT-BIG impact on functional change. Future research should include clinical trials to examine additional external cueing and task-based intervention efficacy with consideration of intensity, frequency, and mode of delivery across disease severity.

## 1. Introduction

Parkinson's disease (PD) is a progressive neurodegenerative disorder affecting the basal ganglia, specifically the dopaminergic neurons of the substantia nigra pars compacta [1, 2]. The basal ganglia play a key role in action selection, and regulation of movement, when impaired, can result in the clinical manifestations of altered movement, specifically tremor, rigidity, postural instability, and bradykinesia [3]. Despite being considered primarily a movement disorder, PD has also been described as a dysfunctional multineurotransmitter pathway disorder [4]. Several nondopaminergic neurotransmitter and neuromodulatory systems in various regions of the central and peripheral nervous system have been implicated in the nonmotor symptoms of Parkinson's disease including but not limited to adenosinergic, noradrenergic, serotonergic, and cholinergic pathways [5]. These additional pathways are thought to contribute to the heterogeneity of symptoms associated with PD symptoms including cognitive impairment, depression and apathy, sensory symptoms such as pain or anosmia, dysautonomia (orthostatic hypotension), and sleep disorders [6]. Ultimately, people with PD (PwPD) experience motor and nonmotor symptoms which may directly impact changes in quality of life and mobility [7, 8].

Research supports exercise interventions for PwPD, to improve activities of daily living and quality of life [9–12]. Multimodal exercise interventions provided by physical therapists (PT) can improve balance, gait, function, and health-related quality of life (HRQoL) [13–15]. Exercise interventions have been shown to facilitate a number of positive outcomes including functional changes and associated neuroplasticity biomarkers in healthy older adults [16] and in PwPD [17, 18]. Exercise increases neuroprotection in animal models through biological mechanisms such as preventing the loss of dopamine cells and increasing neurotrophic factors [19–21]. It is considered neurorestorative because it has been shown to downregulate dopamine transporter thus increasing dopamine in the extracellular space [22], particularly important for PwPD who exhibit decreased dopamine levels [18, 22]. Increased cerebral blood flow, which increases angiogenesis and altered blood brain barrier permeability, is one example of how exercise may provide a component of neuroprotection and promote general brain health [18, 20, 23].

It is important that future development of exercise programming for PwPD is evidence-based and contributes to improved function and quality of life. The American Physical Therapy Association (APTA) Parkinson's Disease EDGE (Evidence Database to Guide Effectiveness) task force published outcome measure recommendations for use in PwPD in clinical and research settings [24]. The APTA task force integrated the International Classification of Functioning, Disability, and Health (ICF) classification levels as outlined by the World Health Organization [25] identified when formulating these recommendations. The ICF model is interactive and identifies three levels of human functioning: (1) the level of body or body part, (2) the whole person, and (3) the whole person in their complete environment. In turn, these levels define three functional dimensions that can be addressed with specific measures: (1) body functions and structures, (2) activities, and (3) participation.

The APTA just recently released clinical practice guidelines for management of PD with recommended modes of exercise including external cueing and task-specific training [26]. The Lee Silverman Voice Treatment BIG (LSVT-BIG) is considered a multimodal intervention because it integrates external cueing and task-based strategies. LSVT-BIG is an exercise training protocol for PwPD [27] that is whole-body, high amplitude, and intensive. Principles of motor learning that drive activity-dependent changes in functional neuroplasticity as outlined by Kleim and Jones have been integrated into the protocol [28]. Although LSVT-BIG is supported as an efficacious treatment in PwPD [26, 27, 29], there has been limited research examining clinically meaningful change in outcome measures across multiple functional domains in PwPD. The purpose of this study was to evaluate individual change across domains of motor function, gait speed, dynamic balance, balance confidence, and quality of life (QoL) in nine participants completing LSVT-BIG at baseline, end of intervention, and 4 weeks after intervention completion. This study is unique due to the integration of PD EDGE recommended measures across multiple domains and the addition of a longitudinal data collection time point which provides potential insight into the timing of exercise-induced functional changes.

## 2. Materials and Methods

A case series that focused on measuring clinically meaningful changes following participation in an attentional strategy training protocol (LSVT-BIG) was conducted with 9 participants (5 male, 4 female). Participant ages ranged from 64 to 76 years, and disease duration ranged from 1 to 17 years. Distribution of disease severity based on Hoehn and Yahr (H&Y) was stage 2 (7 individuals) and stage 3 (2 individuals) (see Table 1).

Participants were recruited from ongoing programs at the University of Rhode Island (URI) and local community. Participants were included in the study if they had a diagnosis of idiopathic PD and no contraindications to exercise. They were excluded from participation if they had uncontrolled cardiovascular disease or history of stroke, other neurologic diagnosis, and/or surgical procedure for treatment of PD including deep brain stimulator. All participants completed informed consent for treatment as approved by the university's Institutional Review Board (1369197-3).

The initial interview was conducted by the principal investigator (PI) and included a comprehensive medical history and demographic data including sex, age, race, education level, current medications, disease severity as measured by H&Y [30], and disease length based on date of medical diagnosis. Additional measures at baseline were collected. These included medical comorbidity in relation to disability as measured by the Cumulative Illness Rating Scale for Geriatrics (CIRS-G) [31], Physical Activity and Disability Survey-Revised (PADS-R) [32] to assess current activity level, and the Montreal Cognitive Assessment (MoCA), using a recommended cut-off score (<26) to detect mild cognitive impairment (MCI) [33] (see Table 1).

### 2.1. Clinical Outcome Measures

The outcome measures for this study were selected from the ICF structure and APTA PD EDGE recommendations. The primary outcome measures were conducted by PI and included (1) *motor function* using the Movement Disorder Society Unified PD Rating Scale (Part III Motor of the MDS-UPDRS) [34], (2) comfortable *gait speed* determined using the 10-meter walk (10MWT) [35], (3) *dynamic balance* using the Mini-Balance Evaluation System Test (MiniBEST) [36, 37], (4) *balance confidence* using Activities-specific Balance Confidence (ABC) scale [38], and (5) health-related *quality of life* (*HRQoL*), measured using the Parkinson's Disease Questionnaire-39 (PDQ-39) [39–41]. These PDQ-39 scores are reported as a health profile summary index score (PDQ-39*SI*) that reflects an overall impact of PD on quality of life [42, 43]. Minimal clinical important difference (MCID), which reflects patient perceptions of improvement, and minimal detectable change (MDC), the detected change that corresponds to a noticeable change in ability, were evaluated for each measure [44, 45] (see Table 2).

### 2.2. Participation and Adherence Measures

A detailed log of the LSVT-BIG protocol was documented by the clinician during the four weeks of treatment. Additionally, an exercise log was maintained by participants during the last four weeks following treatment when they were independently performing their daily home exercises.

### 2.3. Timeline

**Figure 1:**
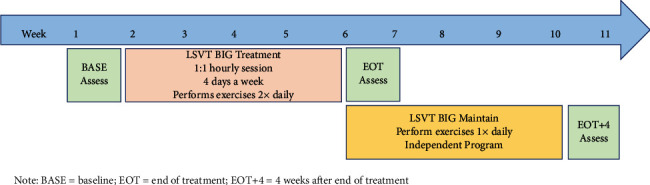
Treatment protocol timeline.

Participants received baseline assessments (BASE), completed the training protocol (four one-hour sessions per week for four weeks of LSVT-BIG), and were instructed to continue their BIG exercises daily for four additional weeks (Figure 1). Assessments were performed during the week following the end of treatment (EOT) and 4 weeks after the end of treatment (EOT+4). All assessments were completed with the participants in an “ON” medication state.

### 2.4. Analysis

High variability of disease symptomology and progression created a concern that clinically relevant effects could be missed during data aggregation if assessing group data; thus, a case series approach was taken. Evidence of individual functional change (improvement or decline) was identified as outcome measures meeting MCID or MCD. Outcome variables including MCID/MCD are provided in Table 2.

The investigators were blind to participant ID and time of collection for data analyses to reduce bias.

### 2.5. Intervention

The external cueing and task-based intervention (LSVT-BIG) was administered for 16 total sessions (individual one-hour sessions, 4× per week × 4 weeks) in the University of Rhode Island's Physical Therapy Clinic. Each session includes exercises, functional and hierarchical task, and carry-over tasks/homework in accordance with the protocol described in Farley and Koshland [27] and Fox et al. [46] The intervention was delivered by certified LSVT-BIG instructors which included two graduate students in the physical therapy clinical doctorate program and the lead PI. The students completed an average of 32 clinical hours administering the intervention prior to participating in treatment for research, and all study personnel were directly supervised by the lead PI.

## 3. Results

### 3.1. Intervention and Compliance

All nine participants completed the research protocol that included evaluations at BASE, the treatment protocol (16 visits), EOT assessments, and EOT+4 assessments. The attrition rate was a 0% although one participant reported a back injury unrelated to the research activity and subsequent pneumonia after completing a baseline assessment one week of treatment. Their participation was placed on hold for two months, and then, a full restart/reassessment was completed. There were no adverse events.

After completion of the 4-week intervention, participants were instructed to continue their exercises once a day as per the LSVT-BIG protocol. All participants kept written daily exercise logs to document compliance during EOT to EOT+4. All participants returned logs at EOT+4 except one. Participants had an 80.3% adherence for these independent exercise sessions, averaging 22.5 sessions out of 28 sessions during this period.

### 3.2. Clinical Measures

**Figure 2:**
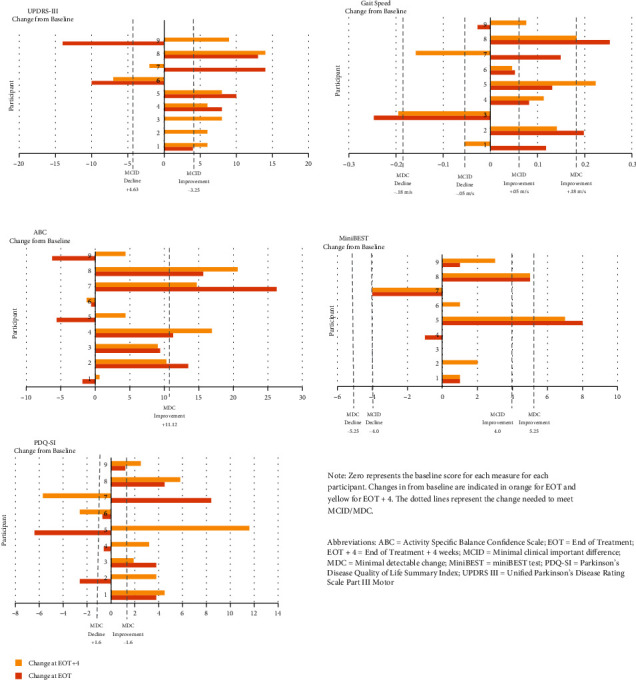
Baseline changes.

Four participants (P2, P4, P5, and P8) demonstrated MCID/MDC in 4 of 5 domains at EOT and EOT+4, and 5 participants demonstrated less robust or less consistent clinical change (see Figure 2).

Clinical improvements, as measured by MCID or MDC, were identified in one or more measures in 8 of the 9 of participants at EOT and EOT+4 across domains of motor function (67%, 78%), gait speed (78%, 67%), balance confidence (44%, 33%), and quality of life (44%, 78%). Only one of the nine participants met MDC and another MCID for the MiniBEST. Only one of the participants (P6), while making improvements across several variables, did not reach MDC for improvement and, in fact, declined in the UPDRS-III and PDQ-SI. MCID/MDC was reached in most participants on the MDS-UPDRS Part III, gait speed, and quality of life measures at the EOT time point, but MCID/MCD was not detected until the EOT+4 time point in three participants on the PDQ-SI and one participant on gait speed. MCID/MCD identified in less than half of participants on measures of balance (ABC and MiniBEST) (see Table 3).

## 4. Discussion

This case series investigated the impact of a multimodal intervention including external cueing and task-based strategies (LSVT-BIG) on motor function, gait speed, dynamic balance, balance confidence, and quality of life in 9 PwPD. Most participants showed clinically significant changes across all domains (MDS-UPDRS Part III, gait speed, ABC, and PDQ-39SI), except dynamic balance (MiniBEST) following completion of intervention (EOT) or 4 weeks following continued independent exercise (EOT+4).

### 4.1. Outcome Measures

A variety of outcome measures as recommended by PD-EDGE across ICF domains, including motor and nonmotor variables, were used to assess a broad spectrum of symptoms that impact those living with PD. The breadth of this analysis distinguishes this case series from previous exercise studies with a limited number of outcome measures focusing almost exclusively on the mobility domain (10 MWT, gait speed).

The primary international rating scale for PD clinical care and research to determine motor disease severity is the MDS-UPDRS [44]. It aligns with the body function and structure on the ICF as it assesses tremor, bradykinesia, and rigidity. In the current study, MCID improvements were identified in MDS-UPDRS III Motor scores in six participants at EOT and seven participants at EOT+4 (see Figure 2) and were consistent with findings from other 8 high-quality and 6 moderate-quality external cueing interventions outlined in the APTA PD CPG [26]. These findings were also consistent with 3 studies focused specifically on LSVT-BIG [29, 47, 48]. One benefit of this measure is it allows comparisons across studies; however, the main drawback is that the MDS-UPDRS requires a trained physician or allied health care professional for administration. Utilizing additional APTA PD EDGE which recommended clinical outcome measures in research across multiple domains such as gait, balance, and quality of life (ICF body function and structures, activities, participation, and personal factors) may provide an improved understanding of the response to treatment and provide practical recommendations to implement in the clinic.

Gait performance under the ICF classification “activities” is represented by variables such as gait speed, step length, and turning speed. Each has been identified as a predictor of morbidity and mortality in PwPD [7, 49], activities of daily living limitations in PwPD [50], and impending disability in older adults [8]. Eight participants had improvements in gait speed at one or more assessment time point, and four of those met MCID and two met MDC at EOT+4 (see Figure 2 and Table 3). These same six participants also met MCID in the UPDRS III Motor score which suggests that external cueing and task-based strategies may potentially decrease the impact of disability in PwPD through reduction in falls and impact on gait speed which is consistent with other similar studies [26, 29, 51].

Gait analysis includes measures that can capture impairments and improvements in PwPD in response to treatment, but gait changes along do not capture the full response to treatment. Postural instability, balance confidence, and fall experience are significant issues for PwPD, yet these measures have been integrated into a limited number of published external cueing and task-based treatment studies [26]. To address this, the current case series included the MiniBEST which is a measure of dynamic balance [37]. While four participants improved in MiniBEST at EOT and six at EOT+4, only one participant reach MDC (P5) and one reached MCID (P8) both at EOT and EOT+4 (see Figure 2 and Table 3).

The positive impact of external cueing and task-based strategies on dynamic balance assessed by the MiniBEST has been supported by two studies [51, 52], both with very small samples sizes (*n* = 2 and *n* = 1, respectively). Previous work identified two subcomponents of the MiniBEST, (1) “anticipatory control” and (2) “sensory orientation,” as not sensitive to improvements.

One consideration for the limited improvement in dynamic balance as measured by MiniBEST may be that the treatment intervention was not task-specific enough regarding balance training. Finally, the ceiling effect of this measure needs to be a consideration in higher functioning PwPD (i.e., P1 and P3), and alternative methods of measuring balance need to be considered when working with this population.

The ABC, a measure of confidence with activities, has been positively correlated with pace-related measures, turning, and dynamic stability during gait in PwPD [52], which are predictors of quality of life and mortality [7]. The findings from the current study found that five participants improved (four met MDC) in the ABC at EOT and eight improved (three maintained MDC) by EOT+4 indicating that the ABC may be a sensitive measure in PwPD following external cueing and task-based interventions (see Figure 2 and Table 3). This is consistent with a previously published LSVT-BIG case study utilizing the ABC to measure balance confidence [53]. Confidence with balance, or lack of confidence, may point toward ICF personal factors that may indirectly drive individuals to move less, demonstrating the importance of measuring and improving this construct in PwPD. Disease severity can impact how closely balance confidence is correlated to actual static and dynamic balance control; thus, results should be interpreted cautiously [54].

Health-related quality of life (HRQoL) is an important consideration for PwPD and represents the ICF participation component. Decreased mobility and function and increased disease severity are associated with impaired HRQoL in PwPD [55]. Four participants met MDC in reported PDQ-39*SI* scores at EOT, and seven met MDC by EOT+4 (see Figure 2 and Table 3). Interestingly, all seven participants met MCID for UPDRS III, and six met MCID/MDC for gait speed. This may indicate how difficult it is to separate how quantitative changes in function may relate to or influence self-reported improvements or decline HRQoL.

Results of this case series identified improvements in ABC and PDQ-SI at the individual level. This may suggest that exercise, especially an intervention utilizing external cueing and task-based interventions that is intensive, may exert slight but important improvements in quality of life and confidence and warrants inclusion of these measures in future research.

### 4.2. Participant Trends

Parkinson's disease is well known for its heterogeneity in symptom onset, disease progression, subtypes, and response to treatment interventions making comparisons within and across research studies extremely challenging. The case series approach in this study allows for assessment of broader trends while also considering individual differences, specifically, length of disease, subtype, and cognitive impairment.

The impact of the external cueing and task-based intervention on function in PwPD appears to trend when time living with PD is taken into consideration, specifically early disease stage (< or = to 5 years since diagnosis) versus mid to late stage (>5 years since diagnosis). Interestingly, participants in the early disease stage (*n* = 5) reached MCD/MCID more consistently (EOT and EOT+4) across more domains. In other words, all 5 participants reached MCD/MCID in 3 (P1), 4 (P2, P4, and P5), or 5 (P8) functional domains. This contrasts with the participants in the mid to late disease stage (*n* = 4) who only reached MCD/MCID in 1 (P6), 2 (P3), 3 (P9), or 4 (P7) functional domains, and these changes had limited consistency (change at EOT and EOT+4). This finding supports the growing evidence that even early in the disease process, there are reductions in amount and intensity of walking [56] and early and regular exercise in PwPD is critical to reducing disability [10, 57]. Mid to late disease stage does not disqualify a PwPD from benefiting from intervention. Despite less consistent change, participants in the mid to late disease stage in this case series still made progress in areas of disease severity, gait speed, and quality of life. This is consistent with studies that have included participants in this disease stage [57]. Moving away from episodic care (treating problems as they arise) and toward a secondary model of prevention (dental model—managing PwPD over the continuum before problems arise) would benefit PwPD earlier in the disease process and perhaps delay the onset of disability compared to current practice [58].

It has been proposed that the postural instability gait disturbance (PIGD) subtype has been associated with more functional disability than tremor dominant (TD) [59] including increased fall risk [60]. The ability to differentiate a patient subtype early in treatment may drive more individualized interventions, improve understanding of patient responsiveness to treatment, and predict disease trajectory [61]. Participants in this case series were categorized as TD, PIGD, or indeterminant subtype based on their UPDRS-III subscores [62]. Of the five participants that were early disease stage, three were TD (P1, P4, and P5) and two (P2, P8) were PIGD but functional outcomes did not appear to be impacted by subtype. Three of the participants (P3, P7, and P9) in the mid to late disease stage were identified as PIGD while the fourth (P6) was considered indeterminant which may have been an additional factor that contributed to the limited changes these individuals realized following the external cueing and task-based intervention. Different clinical measures may be more sensitive or responsive in each subtype. A recent study assessed clinical balance measures as a possible means of directly determining PD subtypes when MDS-UPDRS scores are unavailable and found that the 360-degree turn test was able to distinguish PIGD from TD subtype with high sensitivity using number of steps (>/=7) and time (3.67 seconds) [61]. While this is promising, the 360-degree turn test had low specificity; thus, an additional test addressing reactive postural control is recommended [61]. There is limited research regarding the integration of subtypes into research, and additional subtypes including young onset and rapid progression have been proposed [63]. This case series integrated subtype with mixed results regarding responsiveness to treatment and supports the need to include it in future studies.

Nonmotor symptoms, such as cognitive changes, can also have a significant impact on function and QoL in PwPD. Cognitive impairment in PwPD may represent loss of connectivity within and between brain regions impacted by Lewy body inclusions but can also involve other neurotransmitters such as norepinephrine, serotonin, and acetylcholine [64]. Cognitive impairment can directly impact gait, balance, and quality of life and contribute to increased risk of falls [65], and there was a considerable increased incidence in MCI in individuals identified as PIGD subtype (61.8%) compared with TD subtype (26.8%) [66]. Cross-sectional incidence of MCI in PD has been reported to be 24-31% compared to the global prevalence of 5-7% in the general population of those 60 years and older [64]. The most frequent impaired domain was attention (29.3%) followed by executive function (27.8%) but may also involve visuospatial skills, short- and long-term memory, and language skills such as verbal fluency [64, 66].

The MoCA was administered to screen baseline cognitive skills. All but three of the participants scored at or above the cut-off score of 26. Participant P5 scored a 23 yet had robust functional changes in response to the intervention. Of note is P6 and P7 who each scored a 20, an indication of mild cognitive impairment (MCI). These two participants were able to follow instructions regarding measurements and testing, and each had a supportive care partner that assisted with the follow-through of the home component of the protocol providing additional cueing as needed. Participant P6 had very limited functional changes across the five domains, meeting only MCID for gait speed at EOT and declining in several measures at EOT and EOT+4. This participant (P6) was also mid disease stage and indeterminate subtype possibly combining into a unique set of characteristics that impacted his ability to maximize benefit from the intervention. Participant P7 made gains in four of the domains but only at EOT and then made a clear decline to a level worse than his baseline scores in three of the improved area. They also declined in their perceived balance confidence but did meet MDC at EOT+4. It is worth noting that P7 did experience considerable, albeit intermittent, freezing of gait, and this combined with late disease stage (18 years), PIGD subtype, and cognitive impairment may have made it difficult for them to maintain any gains achieved over the longer period of time when supervision and clinician feedback was removed, and participants were in the independent home component of the protocol.

### 4.3. Timing in Response to Intervention

One important trend identified in the results was the delayed onset of functional changes in response to the external cueing and task-based intervention. The current case series reported individual improvements across most domains from baseline to EOT, but some improvements did not meet MCID/MDC until EOT+4. This may reflect varying time course patterns needed for potential underlying neuroplasticity mechanisms to be impacted before more global functional changes become apparent [67]. Several LSVT-BIG studies that included assessments beyond EOT (specifically EOT+12 weeks) support this concept of continued improvement over time before significant improvements emerge [29, 68].

HRQoL measures have been shown to be resistant to change over a short period of time [9, 57, 69, 70]. The findings from this case series are consistent with only a few individual scores (4, 44%) meeting MDC in the current study at EOT. However, seven participants (78%) went on to report improvements on the PDQ39-SI at EOT+4. Studies that have reported improvements examined interventions that were longer than current intervention (9 and 12 weeks) [71, 72] further supporting the notion that functional changes and the related impact on HRQoL may only be realized over an extended period and support the need for regular follow-up assessments and treatment.

The timing of assessments is important because it highlights the need for consistent research designs in treatment studies to include immediate end of treatment as well as follow-up assessments to (1) understand the time-dependent response to treatment and schedule assessments, accordingly; (2) identify retention of the treatment benefits and time subsequent interventions or assessments to maximize outcome; and (3) compare outcomes across studies for improved translational in the clinic.

### 4.4. Potential Mechanisms

Individual changes in response to external cueing and task-based interventions may include neuroplasticity, motor learning mechanisms, and muscle adaptation. Exercise may serve as a catalyst for neuroplasticity guided by several elements. Frequency, duration, intensity, salience, and setting are all important principles of exercise programs that affect motor learning and driving activity-dependent changes in neural plasticity [28]. External cueing and task-based interventions like LSVT-BIG incorporate each of these principles in treatment to recalibrate amplitude of movement and maintain improvements in mobility following treatment. This intervention is intensive with high frequency and duration (one hour a day, 4 days a week, × 4 weeks). The protocol is also salient as it integrates highly individualized task-based activities that the patient has identified into the functional and hierarchy tasks assigned during treatment (i.e., getting in/out of bed and brushing the teeth). Finally, it provides treatment in a one-to-one delivery model which has been shown to impact outcomes compared to group or independent home programs [69].

Potential mechanisms that underlie these principles of motor learning that may have influenced the changes seen in these PwPD following participation in external cueing and task-based strategies may include the role of automaticity and sensory integration integrated into the intervention. Automaticity, the ability to perform movements without attention directed toward the details of movement, is an important role of the striatum [73]. Bradykinesia, a cardinal motor symptom in PD, reflects a deterioration of motor automaticity. One approach in addressing the deterioration is to integrate increased attention to the activity and cognitive compensatory strategies to maintain or restore movement. External cues and task-based interventions utilize these techniques to drive larger amplitude movements and take advantage of external cues such as auditory (“Move BIG”), visual (the clinician models the movement or provides targets), and tactile (providing passive/active/assistive overpressure to exaggerate or guide movement).

The basal ganglia have been identified as an important area for sensory integration guiding motor planning and execution, and loss of dopamine (i.e., PD) negatively impacts this integration [74]. External cueing interventions utilize techniques that target increased proprioceptive and vestibular input; take advantage of visual, auditory, and tactile cuing; and facilitate improved sensory integration at different intensities throughout the protocol. These types of interventions utilize attention and sensory integration as strategies of motor learning to compensate for deteriorating automaticity early in the protocol to “recalibrate” the smaller and slower movements into larger amplitude and more neurotypical movements by the end of the protocol [73].

Muscle adaptation in response to exercise training can result in improved strength and associated improvements in functional mobility, coordination, and balance. Many PwPD are sedentary and physically inactive, as they tend to fall below age-matched controls for physical activity levels even at time of diagnosis [75]. It is possible that some of the functional changes seen in these participants could be related to the role physical conditioning played in the initial 4 weeks. Two of the four participants (P2, P8) that demonstrated robust functional changes across 4 of 5 domains at EOT and EOT+4 had very low self-reported physical activity on PADS-R which may have contributed to their gains. Yet, two participants (P4, P5) with high self-reported physical activity also had higher, more moderate levels of physical activity and demonstrated robust functional improvements.

It is likely that all these mechanisms contribute to measurable functional changes in the participants in this study in response to an external cueing and task-based intervention. Understanding the role that neuroplasticity, motor learning, and neuromuscular adaptation play in the production of movement is critical to guiding interventions and solutions for people living with movement disorders. Integration of biomarkers should be considered in future research to gather evidence that may link functional changes to underlying neuroplasticity changes. Further research is needed to identify which components of the treatment (i.e., intensity, frequency, tactile cueing, and modelling) and therefore which motor learning principles are most critical for change.

### 4.5. Limitations

This study is not without limitations. First, there was no control or waitlist control group to compare the intervention group. We cannot rule out self-selection bias as participants were not randomly assigned to groups and were aware of the potential benefits of receiving a well marketed external cueing and task-based intervention (LSVT-BIG). There was a small number of participants which is why a case series approach was adopted. Three of the participants scored in the range of MCI on the MoCA (<26), and it is possible that their cognitive status impacted their responsiveness to treatment. Larger studies would need to set inclusion criteria for MoCA scores 26 and above to control for possible cognitive impairment impact on study results.

## 5. Conclusions

The purpose of this study was to evaluate individual changes across domains of motor function, gait speed, dynamic balance, balance confidence, and HRQoL following participation in an external cueing and task-based intervention (LSVT-BIG).

Minimal detectable change (MDC) or minimal clinical important difference (MCID) was observed in one or more measures in 8 of the 9 participants at EOT and EOT+4 and more robustly in four of the five domains in 44% of participants (P2, P4, P5, and P8). Dynamic balance appeared to be the least impacted domain across all but one participant (P5).

The observed individual responses to the external cueing and task-based intervention in this case series add to our understanding of detectable and meaningful changes that can be achieved on measures of motor and nonmotor function. Inclusion of a range of outcome measures as utilized in this case series may help researchers and clinicians assess clinically meaningful change across functional domains in PwPD. Length of disease, subtypes, and cognitive status may directly impact responsiveness to interventions in PwPD and should be considered when designing studies or clinical interventions or assessing responsiveness to treatment. Consideration should be given to the timing of intervention periods and extended assessment period to better detect meaningful change in PwPD following interventions. Exercise, especially those interventions integrating techniques based on principles of motor learning and recommended by the APTA PD Clinical Practice Guidelines, such as external cueing and task-based interventions, should therefore be seen as a critical component in the treatment of individuals with PD.

## Figures and Tables

**Table 1:** Demographic and clinical characteristics.

Participant	Age	Sex	Education	Disease duration	H&Y	UPDRS III baseline	Subtype	Medication LEDD (in mg)	CIRS-G	MOCA	PADS-R
1	65.2	M	1	5	2	15	TD	300	0.80	28	77.40
2	64.8	M	2	3	2	28	PIGD	600	0.60	27	3.80
3	73.2	F	3	16	3	15	PIGD	1050	1.33	26	253.70
4	65.2	F	3	1	2	25	TD	120	2.00	27	123.70
5	74.3	F	3	2	2	26	TD	450	1.86	23	137.00
6	76.3	M	4	7	2	28	Indeterminate	800	1.67	20	34.40
7	65.8	M	4	18	2	50	PIGD	1100	1.86	20	20.60
8	75.8	F	4	4	3	29	PIGD	263	3.80	30	3.80
9	68.8	M	1	9	2	26	PIGD	700	3.50	28	12.60

Average	69.9	5M/4F		7.22	2	26.89	TD	598.11	1.94	25.44	74.11

Range						0-136			0-56	0-30	

Abbreviations: CIRS-G = Cumulative Illness Rating Scale-Geriatric; Education 1 = high school; 2 = some college/technical school; 3 = college; 4 = graduate degree; H&Y = Hoehn and Yahr; LEDD = levodopa equivalent daily; MOCA = Montreal Cognitive Assessment; PADS-R = Physical Activity and Disability Survey-Revised; PIGD = postural instability gait dominant; TD = tremor dominant; UPDRS III = Unified Parkinson's Disease Rating Scale Motor. Note: tremor dominant and postural instability gait disorder subtypes as determined by UPDRS scores; CIRS-G: higher scores indicate higher levels of comorbidity; MOCA: <26 indicates impaired cognition; PADS-R: lower scores are associated with sedentary levels of activity.

**Table 2:** Outcome measure summary with MCID and MDC.

Measure	Domain (ICF)	Range	Direction/interpretation	MDC	MCID	Psychometric properties
ABC [35]	Activity	0-100%	Higher = more confidence	+/- 11.12% [38]	∅	Excellent test-retest (ICC = 0.79) [38] Excellent internal consistency (Cronbach's alpha = 0.92) [38]

Gait speed m/s (Comfortable) [76]	Activity	Continuous variable	Higher/Faster = less impairment < .7 m/s = predictive for falls >1.1 m/s normal walking speed [78]	+/- .18 [49]	+/- .05 [49]	Excellent test-retest (ICC = 0.9) [79] Excellent internal consistency (Cronbach's alpha = 0.9) [79]

10 MWT (Comfortable)	Activity	Continuous variable	Higher/Faster = less impairment	+/- .18 [35]	∅	Excellent test-retest (ICC = 0.96) [35]

MDS-UPDRS III Motor [34]	Activity Body Function Participation	0-132	Lower number = less impairment	∅	-3.25 improve +4.63 worsen [77]	Excellent test-retest (ICC = 0.9) [80] Excellent internal consistency (Cronbach's alpha = 0.93) [34]

MiniBEST [36, 37]	Body Function	0-28	Higher number = less impairment	+/- 5.52 [37]	+/- 4 [81]	Excellent test-retest (ICC = 0.92) [37] Excellent internal consistency (Cronbach's alpha = 0.91) [37]

PDQ39-SI [39, 43]	Participation	0-19.5	Lower number = better quality of life	∅	+/- 1.6 [82]	High test-retest (ICC = 0.68-0.95) [39, 40] Excellent internal consistency (Cronbach's alpha = 0.84-0.94) [42]

Abbreviations: ABC = Activity Specific Balance Confidence Scale; ICF = International Classification of Functioning, Disability, and Health; MCID = Minimal clinical important difference; MDC = Minimal detectable change; MiniBEST = miniBESTest; MWT = meter walk test; PDQ-SI = Parkinson's Disease Quality of Life Summary Index; UPDRS III = Unified Parkinson's Disease Rating Scale Part III Motor. Note: References are indicated in brackets .

**Table 3:** Participant clinical outcomes.

Measure		Participant 1 score (Δ)	Participant 2 score (Δ)	Participant 3 score (Δ)	Participant 4 score (Δ)	Participant 5 score (Δ)	Participant 6 score (Δ)	Participant 7 score (Δ)	Participant 8 score (Δ)	Participant 9 score (Δ)
UPDRS-III	Base	15	28	15	25	26	28	50	29	26
	EOT	*11 (-4)*	29 (1)	*15 (0)*	*17 (-8)*	*16 (-10)*	38 (10)	*36 (-14)*	*16 (-13)*	40 (14)
	EOT+4	*9 (-6)*	*22 (-6)*	*7 (-8)*	*19 (-6)*	*18 (-8)*	35 (7)	52 (2)	*15 (-14)*	*17 (-9)*

MiniBEST	Base	26	24	26	19	15	22	12	13	22
	EOT	27 (1)	24 (0)	26 (0)	18 (-1)	*23 (8)*	22 (0)	8 (-4)	18 (5)	23 (1)
	EOT+4	27 (1)	26 (2)	26 (0)	19 (0)	*22 (7)*	23 (1)	8 (-4)	18 (5)	25 (3)

Gait Speed (m/s)	Base	1.372	0.911	1.524	0.914	0.768	0.951	0.418	0.646	1.301
	EOT	*1.49 (0.12)*	*1.11 (0.2)*	1.28 (-0.25)	*1.0 (0.08)*	*0.9 (0.11)*	*1.0 (0.05)*	*0.57 (0.15)*	*0.9 (0.25)*	1.27 (-0.03)
	EOT+4	*1.32 (-0.06)*	*1.05 (0.14)*	1.33 (-0.2)	*1.03 (0.11)*	*0.99 (0.22)*	1.0 (0.05)	0.26 (-0.16)	*0.83 (0.18)*	*1.38 (0.08)*

ABC	Base	98.75	74.69	87.5	68.75	70	83.75	54.38	49.38	86.88
	EOT	96.88 (-1.88)	*88.13* *(13.44)*	96.88 (9.38)	*80.63* *(11.25)*	64.38 (-5.62)	83.13 (-0.62)	*80.63* *(26.25)*	*65* *(15.63)*	80.63 (-6.25)
	EOT+4	99.38 (0.62)	85 (10.31)	96.56 (9.06)	*69.06* *(16.88)*	74.38 (4.38)	82.5 (-1.25)	*69.06* *(14.69)*	*70* *(20.63)*	91.25 (4.38)

PDQ-SI	Base	9.6	14.7	5.1	15.4	24.4	31.4	35.3	38.5	17.9
	EOT	*5.8 (-3.8)*	17.3 (2.6)	*1.3 (-3.8)*	16 (0.6)	30.8 (6.4)	31.1 (0.7)	*26.9 (-8.4)*	*34 (-4.5)*	16.7 (-1.2)
	EOT+4	*5.1* *(-4.5)*	*10.9* *(-3.8)*	*3.2* *(-1.9)*	*12.2* *(-3.2)*	*12.8* *(-11.6)*	34 (2.6)	41 (5.7)	*32.7* *(-5.8)*	*15.4* *(-2.5)*

Abbreviations: Δ = change from baseline; ABC = Activities-specific Balance Confidence scale; EOT = end of treatment; EOT+4 = end of treatment +4 weeks; MCID = minimal clinical important difference; MDC = minimal detectable change; MiniBEST = MiniBEST test; PDQ-SI = Parkinson's Disease Quality of Life Summary Index; UPDRS III = Unified Parkinson's Disease Rating Scale Part III Motor. Note: italic = improvement meeting minimal clinical important difference (MCID) or minimal detectable change (MCD).

## Data Availability

Data can be made available upon request to the corresponding author.

## References

[1] Little S., Brown P. (2014). Focusing brain therapeutic interventions in space and time for Parkinson’s disease. *Current Biology*.

[2] Rizzi G., Tan K. R. (2017). Dopamine and acetylcholine, a circuit point of view in Parkinson’s disease. *Frontiers in Neural Circuits*.

[3] Kandel E. R., Schwartz J. J. H., Jessell T. M. (2013). *Principles of Neural Science*.

[4] Titova N., Padmakumar C., Lewis S. J. G., Chaudhuri K. R. (2017). Parkinson’s: a syndrome rather than a disease?. *Journal of Neural Transmission*.

[5] Poewe W., Seppi K., Tanner C. M. (2017). Parkinson disease. *Nature Reviews: Disease Primers*.

[6] Jankovic J. (2008). Parkinson’s Disease: Clinical Features and Diagnosis. *Journal of Neurology Neurosurgery and Psychiatry*.

[7] Ellis T., Cavanaugh J. T., Earhart G. M., Ford M. P., Foreman K. B., Dibble L. E. (2011). Which measures of physical function and motor impairment best predict quality of life in Parkinson’s disease?. *Parkinsonism & Related Disorders*.

[8] Ellis T. D., Cavanaugh J. T., Earhart G. M. (2016). Identifying clinical measures that most accurately reflect the progression of disability in Parkinson disease. *Parkinsonism & Related Disorders*.

[9] Speelman A. D., van Nimwegen M., Bloem B. R., Munneke M. (2014). Evaluation of implementation of the ParkFit program: a multifaceted intervention aimed to promote physical activity in patients with Parkinson's disease. *Physiotherapy*.

[10] Oguh O., Eisenstein A., Kwasny M., Simuni T. (2014). Back to the basics: regular exercise matters in Parkinson's disease: results from the National Parkinson Foundation QII registry study. *Parkinsonism & Related Disorders*.

[11] Rafferty M. R., Prodoehl J., Robichaud J. A. (2017). Effects of 2 years of exercise on gait impairment in people with Parkinson disease: the PRET-PD randomized trial. *Journal of Neurologic Physical Therapy*.

[12] Uhrbrand A., Stenager E., Pedersen M. S., Dalgas U. (2015). Parkinson's disease and intensive exercise therapy – a systematic review and meta-analysis of randomized controlled trials. *Journal of the Neurological Sciences*.

[13] Goodwin V. A., Richards S. H., Taylor R. S., Taylor A. H., Campbell J. L. (2008). The effectiveness of exercise interventions for people with Parkinson's disease: a systematic review and meta-analysis. *Movement Disorders*.

[14] Tomlinson C. L., Patel S., Meek C. (2012). Physiotherapy versus placebo or no intervention in Parkinson's disease. *The Cochrane Database of Systematic Reviews*.

[15] Pedrosa D. J., Timmermann L. (2013). Review: management of Parkinson's disease. *Neuropsychiatric Disease and Treatment*.

[16] Voss M. W., Erickson K. I., Prakash R. S. (2013). Neurobiological markers of exercise-related brain plasticity in older adults. *Brain, Behavior, and Immunity*.

[17] Monteiro-Junior R., Cevada T., Oliveira B. R. R. (2015). We need to move more: neurobiological hypotheses of physical exercise as a treatment for Parkinson’s disease. *Medical Hypotheses*.

[18] Petzinger G. M., Fisher B. E., Mcewen S., Beeler J. A., Walsh J. P., Jakowec M. W. (2013). Exercise-enhanced neuroplasticity targeting motor and cognitive circuitry in Parkinson's disease. *Lancet Neurology*.

[19] Petersen A. M. W., Pedersen B. K. (2005). The anti-inflammatory effect of exercise. *Journal of Applied Physiology*.

[20] Voss M. W., Vivar C., Kramer A. F., van Praag H. (2013). Bridging animal and human models of exercise-induced brain plasticity. *Trends in Cognitive Sciences*.

[21] Zigmond M. J., Smeyne R. J. (2014). Exercise: is it a neuroprotective and if so, how does it work?. *Parkinsonism & Related Disorders*.

[22] Hirsch M. A., Iyer S. S., Sanjak M. (2016). Exercise-induced neuroplasticity in human Parkinson's disease: what is the evidence telling us?. *Parkinsonism & Related Disorders*.

[23] Cotman C. W., Berchtold N. C., Christie L. (2007). Exercise builds brain health: key roles of growth factor cascades and inflammation. *Trends in Neurosciences*.

[24] Kegelmeyer D. (2014). Parkinson EDGE task force recommendations: by disease stage (clinical recommendations). http://www.neuropt.org/docs/default-source/parkinson-edge/pdedge-all-documents-combined.pdf?sfvrsn=bccd4f43_2.

[25] World Health Organisation (WHO) (2001). *International Classification of Functioning, Disability and Health*.

[26] Osborne J. A., Botkin R., Colon-Semenza C. (2022). Physical therapist management of Parkinson disease: a clinical practice guideline from the American Physical Therapy Association. *Physical Therapy*.

[27] Farley B., Koshland G. (2005). Training BIG to move faster: the application of the speed–amplitude relation as a rehabilitation strategy for people with Parkinson’s disease. *Experimental Brain Research*.

[28] Kleim J. A., Jones T. A. (2008). Principles of experience-dependent neural plasticity: implications for rehabilitation after brain damage. *Journal of Speech, Language, and Hearing Research: JSLHR*.

[29] Ebersbach G., Ebersbach A., Edler D. (2010). Comparing exercise in Parkinson's disease—the berlin BIG study. *Movement Disorders*.

[30] Hoehn M., Yahr M. (2011). Parkinsonism: onset, progression, and mortality. *Neurology*.

[31] Visser M., Marinus J., Van Hilten J. J., Schipper R. G. B., Stiggelbout A. M. (2004). Assessing comorbidity in patients with Parkinson's disease. *Movement Disorders*.

[32] Kayes N. M., Schluter P. J., McPherson K. M., Taylor D., Kolt G. S. (2009). The Physical Activity and Disability Survey -- Revised (PADS-R): an evaluation of a measure of physical activity in people with chronic neurological conditions. *Clinical Rehabilitation*.

[33] Nasreddine Z. S., Phillips N. A., Bédirian V. (2005). The Montreal Cognitive Assessment, MoCA: a brief screening tool for mild cognitive impairment. *Journal of the American Geriatrics Society*.

[34] Goetz C. G., Tilley B. C., Shaftman S. R. (2008). Movement disorder society-sponsored revision of the unified Parkinson's disease rating scale (MDS-UPDRS): scale presentation and clinimetric testing results. *Movement Disorders*.

[35] Steffen T., Seney M. (2008). Test-retest reliability and minimal detectable change on balance and ambulation tests, the 36-item short-form health survey, and the unified Parkinson disease rating scale in people with parkinsonism. *Physical Therapy*.

[36] Duncan R. P., Leddy A. L., Cavanaugh J. T. (2013). Comparative utility of the BESTest, mini-BESTest, and brief-BESTest for predicting falls in individuals with Parkinson disease: a cohort study. *Physical Therapy*.

[37] Leddy A. L., Crowner B. E., Earhart G. M. (2011). Utility of the Mini-BESTest, BESTest, and BESTest sections for balance assessments in individuals with Parkinson disease. *Journal of Neurologic Physical Therapy: JNPT*.

[38] Powell L. E., Myers A. M. (1995). The activities-specific balance confidence (ABC) scale. *The Journals of Gerontology. Series A, Biological Sciences and Medical Sciences*.

[39] Peto V., Jenkinson C., Fitzpatrick R., Greenhall R. (1995). The development and validation of a short measure of functioning and well being for individuals with Parkinson's disease. *Quality of Life Research*.

[40] Hagell P., Whalley D., Mckenna S. P., Lindvall O. (2003). Health status measurement in Parkinson's disease: validity of the PDQ-39 and Nottingham health profile. *Movement Disorders*.

[41] Hagell P., Nygren C. (2007). The 39 item Parkinson's disease questionnaire (PDQ-39) revisited: implications for evidence-based medicine. *Journal of Neurology, Neurosurgery & Psychiatry*.

[42] Jenkinson C., Fitzpatrick R. A., Peto V. I., Greenhall R., Hyman N. (1997). The Parkinson's Disease Questionnaire (PDQ-39): development and validation of a Parkinson's disease summary index score. *Age and Ageing*.

[43] Peto V., Jenkinson C., Fitzpatrick R. (1998). PDQ-39: a review of the development, validation, and application of a Parkinson's disease quality of life questionnaire and its associated measures. *Journal of Neurology*.

[44] Furlan L., Sterr A. (2018). The applicability of standard error of measurement and minimal detectable change to motor learning research - a behavioral study. *Frontiers in Human Neuroscience*.

[45] Stratford P. W., Riddle D. L. (2012). When minimal detectable change exceeds a diagnostic test–based threshold change value for an outcome measure: resolving the conflict. *Physical Therapy*.

[46] Fox C., Ebersbach G., Ramig L., Sapir S. (2012). LSVT LOUD and LSVT BIG: behavioral treatment programs for speech and body movement in Parkinson disease. *Parkinson's Disease*.

[47] Millage B., Vesey E., Finkelstein M., Anheluk M. (2017). Effect on Gait Speed, Balance, Motor Symptom Rating, and Quality of Life in Those with Stage I Parkinson’s Disease Utilizing LSVT BIG®. *Rehabilitation Research and Practice*.

[48] Ueno T., Sasaki M., Nishijima H. (2017). LSVT-BIG Improves UPDRS III Scores at 4 Weeks in Parkinson’s Disease Patients with Wearing Off: A Prospective, Open-Label Study. *Parkinsons Disease*.

[49] Pulignano G., Del Sindaco D., Di Lenarda A. (2016). Incremental value of gait speed in predicting prognosis of older adults with heart failure: insights from the IMAGE-HF study. JACC. *Heart Failure*.

[50] Tan D., Danoudis M., Mcginley J., Morris M. E. (2012). Relationships between motor aspects of gait impairments and activity limitations in people with Parkinson's disease: a systematic review. *Parkinsonism & Related Disorders*.

[51] Kleppang T. T., Jørgensen L. (2020). Dynamic balance and gait speed improve in persons with Parkinson´s disease after Lee Silverman Voice Treatment (LSVT)-BIG training: a single subject experimental design study. *European Journal of Physiotherapy*.

[52] Henry W., Cline S., Araujo A. C. (2021). Utilizing telehealth to deliver LSVT BIG treatment for young onset Parkinson disease: a case report. *Physical Therapy and Rehabilitation*.

[53] Chatto C. A., York P. T., Slade C. P., Hasson S. M. (2018). Use of a telehealth system to enhance a home exercise program for a person with Parkinson disease: a case report. *Journal of Neurologic Physical Therapy*.

[54] Lee H. K., Altmann L. J., McFarland N., Hass C. J. (2016). The relationship between balance confidence and control in individuals with Parkinson's disease. *Parkinsonism & Related Disorders*.

[55] Schrag A., Jahanshahi M., Quinn N. (2000). How does Parkinson's disease affect quality of life? A comparison with quality of life in the general population. *Movement Disorders: Official Journal of the Movement Disorder Society*.

[56] Cavanaugh J. T., Ellis T. D., Earhart G. M., Ford M. P., Foreman K. B., Dibble L. E. (2012). Capturing ambulatory activity decline in Parkinson's disease. *Journal of Neurologic Physical Therapy*.

[57] Schenkman M., Hall D. A., Barón A. E., Schwartz R. S., Mettler P., Kohrt W. M. (2012). Exercise for people in early- or mid-stage Parkinson disease: a 16-month randomized controlled trial. *Physical Therapy*.

[58] Ellis T. D., Colón-Semenza C., DeAngelis T. R. (2021). Evidence for early and regular physical therapy and exercise in Parkinson's disease. *Seminars in Neurology*.

[59] Jankovic J., McDermott M., Carter J. (1990). Variable expression of Parkinson's disease: A base‐line analysis of the DAT ATOP cohort. *Neurology*.

[60] Pelicioni P. H., Menant J. C., Latt M. D., Lord S. R. (2019). Falls in Parkinson’s disease subtypes: risk factors, locations and circumstances. *International Journal of Environmental Research and Public Health*.

[61] Prime M., McKay J. L., Bay A. A. (2020). Differentiating Parkinson disease subtypes using clinical balance measures. *Journal of Neurologic Physical Therapy*.

[62] Stebbins G. T., Goetz C. G., Burn D. J., Jankovic J., Khoo T. K., Tilley B. C. (2013). How to identify tremor dominant and postural instability/gait difficulty groups with the movement disorder society Unified Parkinson's Disease Rating Scale: comparison with the Unified Parkinson's Disease Rating Scale. *Movement Disorders*.

[63] Marras C., Lang A. (2013). Parkinson's disease subtypes: lost in translation?. *Journal of Neurology, Neurosurgery, and Psychiatry*.

[64] Dag A., Lucia B., Halliday G. M. (2021). Parkinson disease-associated cognitive impairment. *Nature Reviews: Disease Primers*.

[65] Latt M. D., Lord S. R., Morris J. G. L., Fung V. S. C. (2009). Clinical and physiological assessments for elucidating falls risk in Parkinson's disease. *Movement Disorders*.

[66] Monastero R., Cicero C. E., Baschi R. (2018). Mild cognitive impairment in Parkinson’s disease: the Parkinson’s disease cognitive study (PACOS). *Journal of Neurology*.

[67] Miller N., Noble E., Jones D., Burn D. (2006). Life with communication changes in Parkinson’s disease. *Age and Ageing*.

[68] Dashtipour K., Johnson E., Kani C. (2015). Effect of exercise on motor and nonmotor symptoms of Parkinson’s disease. *Parkinson’s Disease*.

[69] King L. A., Wilhelm J., Chen Y. (2015). Effects of group, individual, and home exercise in persons with Parkinson disease: a randomized clinical trialdisease. *Journal of Neurologic Physical Therapy*.

[70] Ridgel A. L., Vitek J. L., Alberts J. L. (2009). Forced, not voluntary, exercise improves motor function in Parkinson's disease patients. *Neurorehabilitation and Neural Repair*.

[71] Cugusi L., Solla P., Zedda F. (2014). Effects of an adapted physical activity program on motor and non-motor functions and quality of life in patients with Parkinson's disease. *NeuroRehabilitation*.

[72] Combs S. A., Diehl M. D., Chrzastowski C. (2013). Community-based group exercise for persons with Parkinson disease: a randomized controlled trial. *NeuroRehabilitation*.

[73] Wu T., Hallett M., Chan P. (2015). Motor automaticity in Parkinson's disease. *Neurobiology of Disease*.

[74] Konczak J., Corcos D. M., Horak F. (2009). Proprioception and motor control in Parkinson's disease. *Journal of Motor Behavior*.

[75] Ellis T., Rochester L. (2018). Mobilizing Parkinson's disease: the future of exercise. *Journal of Parkinson's Disease*.

[76] Curtze C., Nutt J. G., Carlson-Kuhta P., Mancini M., Horak F. B. (2016). Objective gait and balance impairments relate to balance confidence and perceived mobility in people with Parkinson disease. *Physical Therapy*.

[77] Horváth K., Aschermann Z., Ács P. (2015). Minimal clinically important difference on the motor examination part of MDS-UPDRS. *Parkinsonism & Related Disorders*.

[78] Montero-Odasso M., Schapira M., Soriano E. R. (2005). Gait velocity as a single predictor of adverse events in healthy seniors aged 75 years and older. *The Journals of Gerontology Series A: Biological Sciences and Medical Sciences*.

[79] Studenski S., Perera S., Wallace D. (2003). Physical performance measures in the clinical setting. *Journal of the American Geriatrics Society*.

[80] Siderowf A., McDermott M., Kieburtz K. (2002). Test–retest reliability of the Unified Parkinson's Disease Rating Scale in patients with early Parkinson's disease: results from a multicenter clinical trial. *Movement Disorders*.

[81] Godi M., Arcolin I., Giardini M., Corna S., Schieppati M. (2020). Responsiveness and minimal clinically important difference of the mini-BESTest in patients with Parkinson’s disease. *Gait & Posture*.

[82] Peto V., Jenkinson C., Fitzpatrick R. (2001). Determining minimally important differences for the PDQ-39 Parkinson's Disease Questionnaire. *Age and Ageing*.

[83] Clarkin C. M. (2020). LSVT^®^BIG exercise-induced neuroplasticity in people with Parkinson’s disease: an assessment of physiological and behavioral outcomes. https://digitalcommons.uri.edu/oa_diss/1151.

